# Realisation of topological zero-energy mode in bilayer graphene in zero magnetic field

**DOI:** 10.1038/s41598-017-06902-9

**Published:** 2017-07-25

**Authors:** Janghee Lee, Kenji Watanabe, Takashi Taniguchi, Hu-Jong Lee

**Affiliations:** 1 Department of Physics, Pohang University of Science and Technology, Pohang, 790-784 Republic of Korea; 2 National Institute for Materials Science, Namiki 1-1, Tsukuba, Ibaraki 305-0044 Japan

## Abstract

Bilayer graphene (BLG) gapped by a vertical electric field represents a valley-symmetry-protected topological insulating state. Emergence of a new topological zero-energy mode has been proposed in BLG at a boundary between regions of inverted band gaps induced by two oppositely polarized vertical electric fields. However, its realisation has been challenged by the enormous difficulty in arranging two pairs of accurately aligned split gates on the top and bottom surfaces of clean BLG. Here we report realisation of the topological zero-energy mode in ballistic BLG, with zero-bias differential conductance close to the ideal value of 4 *e* ^2^/*h* (*e* is the electron charge and *h* is Planck’s constant) along a boundary channel between a pair of gate-defined inverted band gaps. This constitutes the *bona fide* electrical-gate-tuned generation of a valley-symmetry-protected topological boundary conducting channel in BLG in zero magnetic field, which is essential to valleytronics applications of BLG.

## Introduction

Ever since the topological invariant was first identified in the quantum Hall effect^1, 2^, many efforts have been made to explore new topological phases in condensed matter. The efforts have led to the recent discovery of a time-reversal-symmetric topological phase^3^ (*Z*_2_-topology) in two^4, 5^ and three dimensions^6, 7^. Emergence of a new one-dimensional (1D) valley-symmetry-protected topological zero-energy mode has recently been proposed in bilayer graphene (BLG)^8–10^ at a boundary between regions of inverted band gaps opened by a pair of oppositely polarized vertical electric fields. However, its realisation has been challenged by the enormous difficulty in arranging two pairs of accurately aligned split gates on the top and bottom surfaces of clean BLG. Here we accurately confirm the emergence of the valley-symmetry-protected topological phase in BLG. Encapsulation of a BLG layer within two hexagonal boron nitride crystals (hBNs) led to ballistic transport of carriers in our devices. We introduced novel schemes for fabricating vertically aligned four split gates attached onto top and bottom hBNs. By fine-tuning external electric field in each region in the BLG in opposite polarities, in the absence of an external magnetic field, we observed metallic conduction even with each region of the BLG in insulating state. The zero-bias conductance along the boundary between two insulating regions was very close to the theoretical prediction, 4 *e* ^2^/*h* (*e* is the electron charge and *h* is Planck’s constant). Current-voltage characteristics comparing with the numerical calculation for the topologically trivial bound states formed at the band-inversion boundary reveal that the zero-bias differential conductance is attributed to the topological zero-energy states confined at the boundary. This confirms the electrical-gate-tuned realisation of a valley-symmetry-protected topological boundary channel in BLG^8–11^ in zero magnetic field, which affords the essential building blocks of valleytronic applications and valley-associated functionalities.

## Results

### The zero-energy states in BLG

The low-energy state of intrinsic BLG, Bernal-stacked two monolayer graphene sheets, can be approximated by massive chiral quasiparticle bands without a band gap as the leading term of the interlayer coupling is considered in the Hamiltonian of BLG; however, a band gap is induced and tuned by the broken inversion symmetry in an external electric field applied perpendicular to the plane of a BLG layer^12, 13^. The gate-tunability of the band gap and carrier density of BLG has been conveniently utilized for effective carrier confinement^14–17^ and has led to a theoretical proposition for a new type of symmetry-protected topological one-dimensional (1-D) channel of zero-energy mode in BLG^8, 9, 11, 18^.

**Figure 1:**
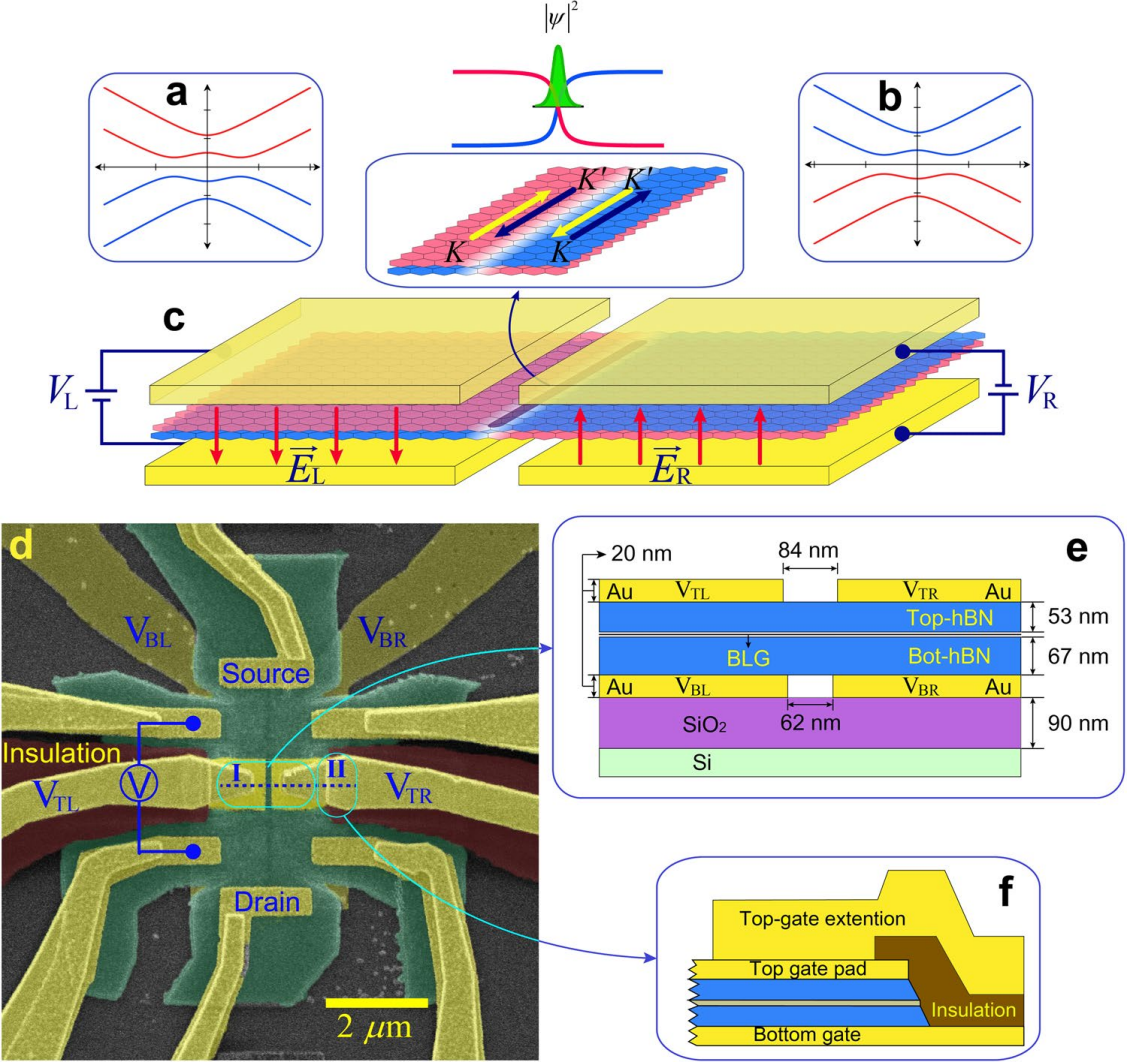
Topological zero-energy mode in bilayer graphene. (**a**,**b**) Gapped band structures of BLG corresponding to the (**a**) left and (**b**) right half. (**c**) Schematic illustration of the topological zero-energy mode in BLG. Different colours in **a** and **b** indicate opposite chiralities. The schematic highlighted view in **c** illustrates the electric current direction of each valley-momentum-locked zero-energy mode at the boundary between two oppositely dual-gated regions in BLG. (**d**) False-coloured scanning electron microscopy (SEM) image of the four-gated device. (**e**,**f**) Cross-sectional view along the dotted line in region I and II in **d**, respectively (see Method and Supplementary Fig. 7 for details).

Figure 1a,b and c illustrate schematically how the topological zero-energy mode emerges in BLG. A vertical electric field ${\overrightarrow{E}}_{{\rm{L}}}({\overrightarrow{E}}_{{\rm{R}}})$ generated by the left (right) gate voltage *V*_L_ (*V*_R_) induces a band gap on the left (right) side of the BLG layer (Fig. 1a and b). For opposite polarities of ${\overrightarrow{E}}_{{\rm{L}}}$ and ${\overrightarrow{E}}_{{\rm{R}}}$, the band gap closes and reopens with inverted chirality at the boundary between the two gapped BLG regions. Thus, the gapless boundary is topologically protected in the absence of valley-mixing perturbations^8, 10^. There are two 1-D valley-momentum-locked zero-energy states with opposite chirality for each valley (Fig. 1c). Thus, the zero-bias differential conductance (*dI*/*dV*) along the boundary has a quantized conductance of 4 *e* ^2^/*h* as long as inter-valley scattering is absent. Here, the factor of 4 comes from the spin degeneracy and the two copropagating modes in a valley in the current direction. Because the symmetry-protected topological zero-energy mode in BLG is vulnerable to valley-mixing perturbations^19^, prevention of inter-valley scattering is key to demonstrating the topological 1-D mode with ideal 4 *e* ^2^/*h* conductance. Conductance values falling short of 4 *e* ^2^/*h* observed in a previous study^20^ for an AB-BA-stacked domain boundary in BLG were caused by valley-mixing scattering with diffusive transport in the device. Also a recent report on the zero-bias conductance in split dual-gated BLG^21^ similar to ours showed much smaller values (~0.5 *e* ^2^/*h*) than the prediction by disorder-induced high backward scattering at the domain boundary. In the study, the conductance of ~4 *e* ^2^/*h* were attained only when the 1-D backward scattering was suppressed in a high magnetic field. In this case, however, the quantized conductance of the ordinary quantum Hall edge transport may have contributed to the intended boundary-channel conductance. Thus, to clearly confirm the formation of the valley-symmetry-protected topological 1-D zero-energy mode at the boundary between the inverted band-gap regions in BLG, observation of the conductance of 4 *e* ^2^/*h* is highly required without applying a high magnetic field.

### Zero-bias conductance along the kink-potential

Figure 1d is a false-coloured scanning electron microscopy (SEM) image of our device. The extension lead of the left (right) split bottom gate is denoted as V_BL_ (V_BR_). Figure 1e (f) shows a schematic cross-sectional view of the narrow corridor along the dotted line in region I (II) of Fig. 1d. The dimensions in Fig. 1e were determined by SEM and atomic force microscopy (AFM)^22, 23^. A negative resistance in the van der Pauw configuration confirms the ballistic transport of carriers within the device size (see Supplementary Fig. 1).

**Figure 2:**
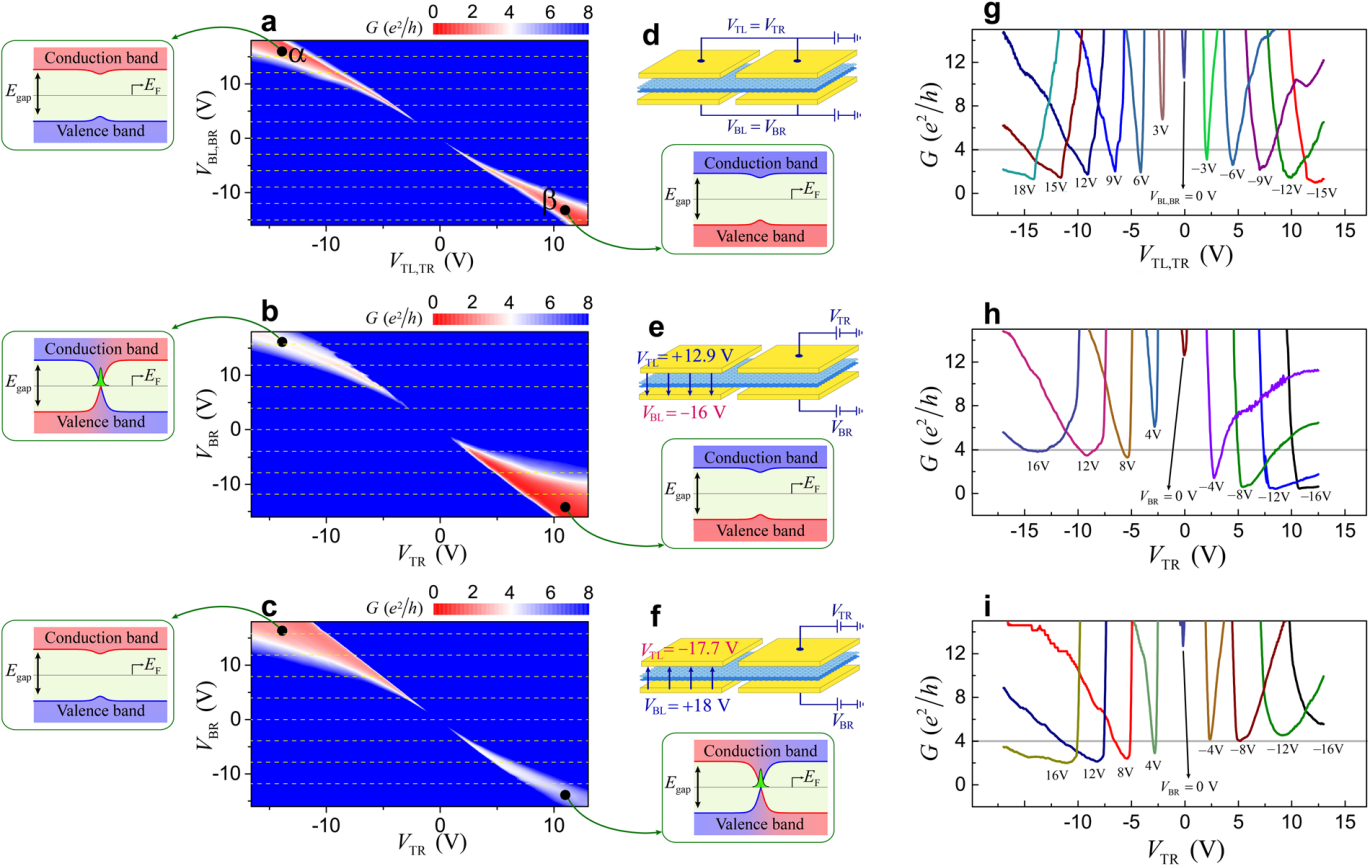
Symmetric- and asymmetric-gate configurations. (**a**) *V*_TL,TR_ and *V*_BL,BR_ (**b**,**c**) (*V*_TR_ and *V*_BR_) dependence of the conductance (*G*) in coded colours. The white conductance region corresponds to the value of *G* = 4 *e* ^2^/*h*. Each rounded box illustrates the band gap profile of the corresponding conductance region along the dotted line in the region I in Fig. 1d. (**d**–**f**) Schematic measurement configuration. (**g**–**i**) Slice traces corresponding to dashed lines in the conductance maps in **a**–**c**. The horizontal solid lines are the quantum conductance of *G* = 4 *e* ^2^/*h*.

Figures 2a–c show zero-bias *dI*/*dV* obtained for the measurement configurations shown in Fig. 2d–f, respectively, for different bottom- and top-gate voltages, *V*_BL_, *V*_BR_, *V*_TL_, and *V*_TR_. Transport measurements were made using the standard lock-in technique with a frequency of 17.77 Hz at 0.3 K. We define the symmetric-gate (asymmetric-gate) configuration as that where a pair of top and bottom split gates are connected to the respective voltage sources, as shown schematically in Fig. 2d (e and f).

The colour map of Fig. 2a shows the *V*_TL,TR_ and *V*_BL,BR_ gate-voltage dependence of the zero-bias *dI*/*dV* in the symmetric-gate configuration. A typical feature of this dual-gated BLG is clearly seen in Fig. 2a with suppressed conductance (insulating behaviour, see Supplementary Note 1) at the upper-left and lower-right regions, even with a narrow corridor that was not covered by top and bottom gates at the centre of the device (see Fig. 1). The gating effect of the split gates was maintained across the corridor with little degradation compared with dual-gated BLG (see Method and Supplementary Fig. 2). Following the convention of the previous study^12^, we define the displacement field as ${\overrightarrow{D}}_{{\rm{T}}}=-{\varepsilon }_{{\rm{hBN}}}\frac{{V}_{{\rm{T}}}-{V}_{{\rm{T}}}^{0}}{{d}_{{\rm{T}}}}\hat{z}$ and ${\overrightarrow{D}}_{{\rm{B}}}={\varepsilon }_{{\rm{hBN}}}\frac{{V}_{{\rm{B}}}-{V}_{{\rm{B}}}^{0}}{{d}_{{\rm{B}}}}\hat{z}$, where, *ε*_*hBN*_ is the dielectric constant of hBN^22^ (~3.9), *d*_*T *_(*d*_*B*_) is the thickness of the top (bottom) hBN, ${V}_{{\rm{T}}}^{0}({V}_{{\rm{B}}}^{0})$ is the charge neutrality point (CNP) of *V*_T _(*V*_B_), and $\hat{z}$ is the unit vector normal to the BLG sheet. From Fig. 2a, the CNP was determined to be $({V}_{{\rm{T}}}^{0},\,{V}_{{\rm{B}}}^{0})=(-0.7\,{\rm{V}},1\,{\rm{V}})$. The magnitude of the average displacement field, $|\overrightarrow{D}|=|({\overrightarrow{D}}_{{\rm{T}}}+{\overrightarrow{D}}_{{\rm{B}}})/2|$, determines the size of the band gap (*E*_gap_), and the total carrier density in BLG is estimated by $n=\frac{{{\rm{\varepsilon }}}_{0}}{e}({\overrightarrow{D}}_{{\rm{B}}}-{\overrightarrow{D}}_{{\rm{T}}})\cdot \hat{z}$. Along the diagonal track from region ‘*α*’ to ‘*β*’ in Fig. 2a, the Fermi level (*E*_F_) is maintained in the middle of the *E*_gap_ while *E*_gap_ increases away from the CNP.

Figure 2b shows the conductance for an asymmetric-gate configuration with fixed *V*_TL_ (=12.9 V) and *V*_BL_ (=−16 V), while *V*_TR_ and *V*_BR_ were varied as shown in Fig. 2e. Thus, the left region of the BLG was fixed in an insulating state (at the region *β* in Fig. 2a), while the right region covered the entire state in Fig. 2a. Device conductance in region *α* is drastically enhanced in Fig. 2b, although the right region in the BLG remains in an insulating state similar to the left half. The difference in the upper left region of Figs. 2a and b results from inverted relative polarity between ${\overrightarrow{D}}_{{\rm{L}}}$ (left region) and ${\overrightarrow{D}}_{{\rm{R}}}$ (right region), i.e. depending on whether ${\overrightarrow{D}}_{{\rm{L}}}\cdot {\overrightarrow{D}}_{{\rm{R}}} > 0$ or ${\overrightarrow{D}}_{{\rm{L}}}\cdot {\overrightarrow{D}}_{{\rm{R}}} < 0$. This indicates the emergence of a topological conducting channel between the two inversely gapped regions. This feature was reproduced in another measurement configuration of Fig. 2f, plotted in Fig. 2c using the same colour scheme. In this case, the set of values for *V*_TL_ and *V*_BL_ were fixed at (−17.7 V, 18 V) while *V*_TR_ and *V*_BR_ were varied. Compared with Fig. 2b, the positions of the insulating (red) and conducting (white) regions were reversed.

The common features in Fig. 2a–c are as follows. The device exhibits insulating behaviour for ${\overrightarrow{D}}_{{\rm{L}}}\cdot {\overrightarrow{D}}_{{\rm{R}}} > 0$ (red region) and conducting behaviour for ${\overrightarrow{D}}_{{\rm{L}}}\cdot {\overrightarrow{D}}_{{\rm{R}}} < 0$ (white region) although both regions in the BLG remain in the insulating state by themselves. Figure 2g (h,i) shows slice traces corresponding to the dashed lines in Fig. 2a (b,c) for given values of *V*_BL,BR_ (*V*_BR_). It is clear that the minimum conductance in each trace (corresponding to a charge neutral state in the right region) approaches 4 *e* ^2^/*h* of the valley-symmetric topological mode (horizontal solid lines) only for ${\overrightarrow{D}}_{{\rm{L}}}\cdot {\overrightarrow{D}}_{{\rm{R}}} < 0$.

### Current-voltage characteristics of the 1-D conducting channel in BLG

Topologically trivial (non-chiral) bound states also form at the potential well (kink-potential) along the corridor. However, these bound states are separated by a finite energy from *E*_F_, which remains in the middle of the band gap along the diagonal track in Fig. 2a. Thus, the zero-bias *dI*/*dV* plotted in Fig. 2a–c is attributed to the zero-energy state formed at the kink-potential well. This fits with the theoretical prediction of valley-symmetry-protected topological zero-energy mode in the BLG^8, 10^.

**Figure 3:**
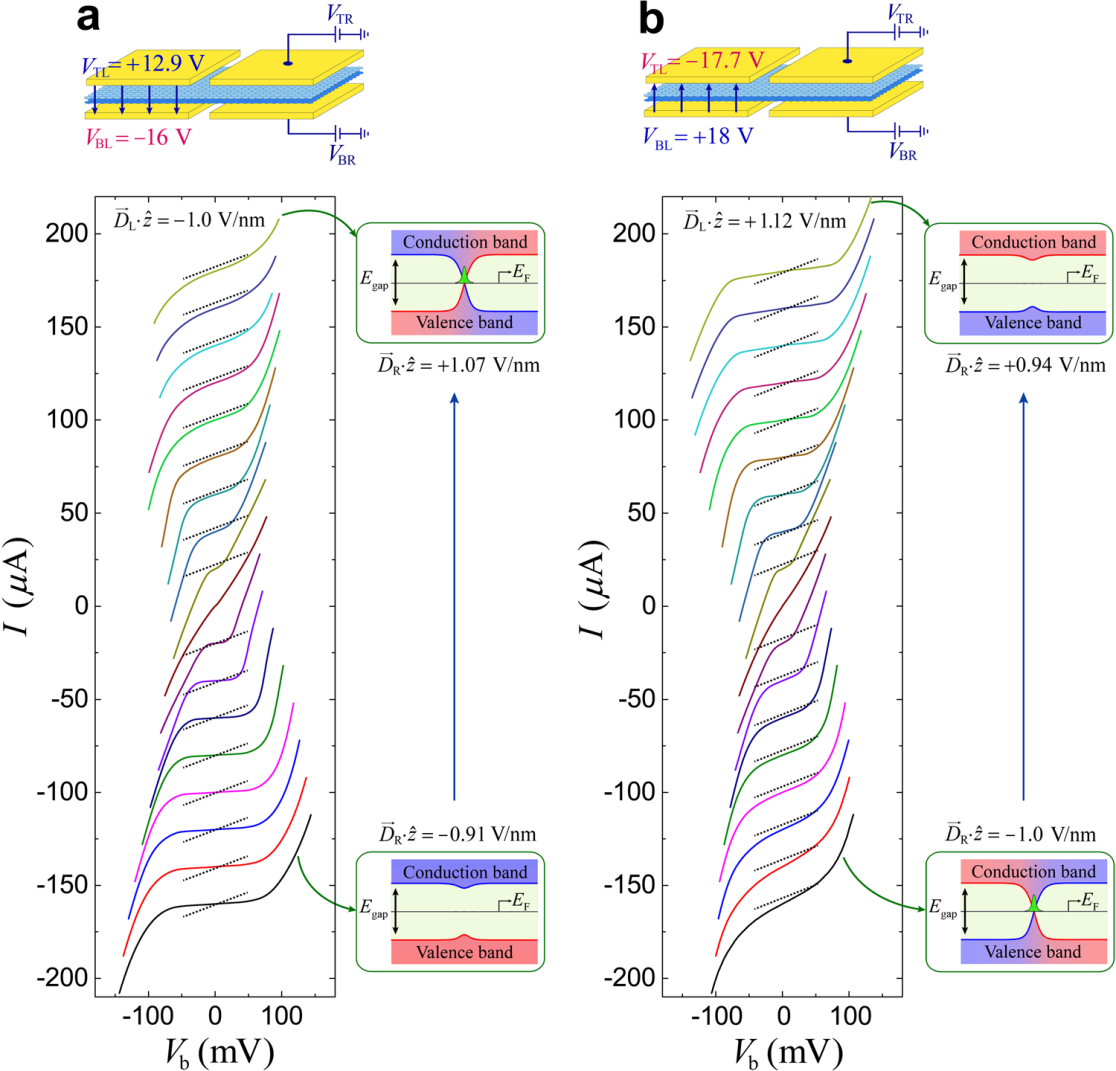
Current–voltage (*I*-*V*) characteristics in asymmetric-gate configurations. (**a**,**b**) *I*–*V* curves obtained at different points along the diagonal track (from *α* to *β*) in (**a**) Fig. 2b and (**b**) Fig. 2c, respectively. Upper illustrations represent measurement configurations for the two sets of *I*–*V* curves. Dotted lines correspond to the slope of 4 *e* ^2^/*h*.

This assertion is more clearly justified by examining the current–voltage (*I*–*V*) characteristics of the 1-D conducting channel along the corridor for ${\overrightarrow{D}}_{{\rm{L}}}\cdot {\overrightarrow{D}}_{{\rm{R}}} > 0$ and ${\overrightarrow{D}}_{{\rm{L}}}\cdot {\overrightarrow{D}}_{{\rm{R}}} < 0$, with *E*_F_ fixed in the middle of the band gap. Figure 3a (b) shows *I*–*V* curves obtained for the asymmetric-gate configuration illustrated in Fig. 2e (f). For the measurements, the value of ${\overrightarrow{D}}_{{\rm{L}}}\cdot \hat{z}$ was fixed at −1.0 V/nm (+1.12 V/nm) while that of ${\overrightarrow{D}}_{{\rm{R}}}\cdot \hat{z}$ was varied from positive (the region *α* in Fig. 2a) to negative (the region *β* in Fig. 2a). Each trace in Fig. 3a and b is shifted vertically for clarity. The dotted lines indicate 4 *e* ^2^/*h*, to highlight the difference in the zero-bias slope for ${\overrightarrow{D}}_{{\rm{R}}}\cdot \hat{z} > 0$ and ${\overrightarrow{D}}_{{\rm{R}}}\cdot \hat{z} < 0$. The band gap profiles along the dotted line in region I of Fig. 1d are illustrated for the corresponding *I*–*V* curves denoted by the arrows.

For ${\overrightarrow{D}}_{{\rm{L}}}\cdot {\overrightarrow{D}}_{{\rm{R}}} > 0$, *I*–*V* curves exhibit the typical nonlinear feature of the insulating state with a finite transport gap, which increases with *E*_gap_. Although *I*–*V* curves for ${\overrightarrow{D}}_{{\rm{L}}}\cdot {\overrightarrow{D}}_{{\rm{R}}} < 0$ also show nonlinearity for high biases, the high zero-bias *dI*/*dV* (~4 *e* ^2^/*h*) cannot be associated with the insulating state of the BLG. It can be explained only by assuming a conducting channel at zero energy. The slope of *I-V* for ${\overrightarrow{D}}_{{\rm{L}}}\cdot {\overrightarrow{D}}_{{\rm{R}}} < 0$ increases for high values of *V*_b_ as additional conducting channels existing at finite energies participate in conduction.

The increase of channel conductance in the higher bias range in Fig. 3 is due to the emergence of the topologically trivial additional transverse conducting modes that are confined in the kink potential well along the corridor. Calculation of the energy levels in the kink potential based on the full structure of the Hamiltonian in BLG has been reported previously^18, 21^. It predicts that the energy of the lowest bound level of the trivial conducting state is a few tens of meV. In fact, in Fig. 3, the observed differential conductance *dI*/*dV* starts to increase abruptly for the bias voltage *V*_b_ above a certain threshold value larger than ~10 meV. We believe that the threshold voltage corresponds to the lowest bound energy level *ε*_1_ of the topologically trivial modes. Thus, the trivial conducting channels are distinctly separated from the topological zero-energy mode and the zero energy conductance observed in this study corresponds entirely to the topological conducting modes predicted theoretically. However, the conductance in Fig. 3 increases without any step-like features at the quantized eigenenergies of the trivial conducting levels, *ε*_*n*_. This feature arises because transport in the topologically trivial non-chiral bound states along the narrow conducting channel was not necessarily ballistic, as these states were not protected from non-valley-mixing perturbations, such as long-range scattering. The rapid increase of the conductance for high biases is due to conduction through the continuum state. The probing voltage level for the conductance map in Fig. 2 is ~0.5 mV which is far less than the lowest bound-state level of ~10 mV for the trivial bound states. The chiral zero-energy modes are clearly differentiated from the contribution of the topologically trivial non-chiral conducting channels.

An analysis in association with another four-gated device indicates that the precise alignment of two pairs of split gates within a few nanometres is of prime importance for observing topological 1-D conduction. In addition, controlling the thicknesses of the bottom and top hBN layers as close as possible is also essential, as the thickness ratio of the two hBN layers governs the symmetry of the top and bottom gating (see Supplementary Figs 3–5 and Supplementary Note 2). The thickness ratio was ~1.26 for the device in Fig. 1d.

## Discussion

The zero-energy conducting mode observed in this study is robust as long as the valley symmetry is conserved along the corridor, similar to the robustness of the chiral edge mode protected by the time reversal symmetry in a quantum spin Hall insulator. The BLG layer in our device, encapsulated by two clean hBN single crystals, leads to the ballistic transport within the device size as shown in Supplementary Fig. 1. In addition, since the conducting channel is established inside the BLG away from the atomically rugged edge, valley-mixing scattering is suppressed significantly^24^. Although there may be some scattering sources such as localized states confined in the kink-potential corridor, the energy levels of those confined states are located sufficiently away from the zero energy and thus the ZBDC is hardly affected by them.

In our device, two ends of the corridor are connected to each wide region of BLG encapsulated by two hBN layers with only bottom gate. Therefore, in these two wide regions of BLG, the Fermi levels are located at the conduction bands with ballistic transport. In this case, the wide regions of the BLG become the electron reservoirs, taking a role of source and drain contacts to the 1-D conducting channel at the corridor. According to the Landauer-Buttiker formalism, the conductance of a 1-D conducting channel is determined by the potential drop at the interfaces between the reservoirs and the constricted 1-D channel. Thus, the potential drop between the wide BLG regions, on the sides of source and drain, and the corridor in our device are already included in the measured channel conductance.

The manipulation of the valley degrees of freedom, which is at the core of this study, in various two-dimensional (2-D) materials has attracted enormous attention due to their possible utilization in dissipationless valleytronics applications. However, most previous studies on the subject have focused on optical manipulation. The study of transport based on the valley degrees of freedom is still in its infancy; a valley-specific transport signal has been reported only for non-local measurements in Hall-bar-type devices^25, 26^. The 1-D topological carrier guiding demonstrated in this study affords a promising route to valleytronic applications and sophisticated valley associated functionalities based on 2-D materials.

## Methods

### Stacking procedure

For device preparation, we first encapsulated a BLG sheet between two atomically clean hexagonal boron nitride (hBN) single crystals^22, 23, 27^. A Gel-film (Gel-Pak, PF-30/17-X4) was attached to a slide glass, to be used as a stamp. The hBN flakes (bottom hBN) were then mechanically exfoliated onto the Gel-film using the ‘Scotch tape’ method^27^. The prepared stamp (bottom hBN/Gel-film/slide glass) was affixed to an optical microscope to pick up a piece of BLG, which was transferred onto a highly doped silicon wafer capped with 90-nm-thick SiO_2_ (SiO_2_/Si). After picking up the BLG, the assembled stack (bottom hBN/BLG) was deposited onto the target hBN (top hBN), which had been exfoliated onto another SiO_2_/Si substrate coated with double layers of polymer film consisting of water soluble poly(4-styrene sulfonic acid) (PSS) and poly(methyl methacrylate) (PMMA). Then the prepared substrate was floated on deionised water, which dissolves the PSS layer. When the SiO_2_/Si substrate was detached from the PMMA film, which supported the hBN/BLG/hBN heterostructure, the PMMA membrane was affixed to an optical microscope to transfer the assembled structure onto the split bottom gates patterned on another SiO_2_/Si substrate using standard electron-beam lithography (see Supplementary Fig. 6).

### Patterning procedure

After patterning the split bottom gates, which consisted of Cr/Au (5 nm/15 nm) double layers, the hBN/BLG/hBN heterostructure was transferred onto the double layers within sub-micrometre accuracy by a similar technique to that used in the stacking processes. After patterning the bottom-gate extension leads, electrical contacts to the BLG were made by atomic edge contact, which is a method modified from a previous study^23^ (without removal of the bottom hBN). The pair of split-gate pads (1.5 × 1 μm^2^) consisting of Cr/Au (3 nm/12 nm) double layers were then deposited onto the top hBN with lateral positions aligned within a few nm accuracy by electron-gun evaporation together with electron-beam nanofabrication. During the CF_4_/O_2_ plasma etching of the device geometry, each boundary of the two split dual-gated regions in the BLG was removed using the pair of split top gates as etching stencils. To prevent electrical shortage between the BLG and top-gate extension leads, the edge of the BLG was covered with double insulating layers (120 nm-thick Al_2_O_3_ and 130-nm-thick cross-linked PMMA), followed by electron-gun evaporation of the top-gate extension leads. See Supplementary Fig. 7 for details and image for each step.

## Electronic supplementary material

Supplementary Information
