# Supplementary material for: Realisation of topological zero-energy mode in bilayer graphene in zero magnetic field
Source: Sci Rep. 2017 Jul 25;7:6466. doi: 10.1038/s41598-017-06902-9 (PMC5527089; doi:10.1038/s41598-017-06902-9)
Supplement: Supplementary file 1 — Supplementary Information [file 41598_2017_6902_MOESM1_ESM.pdf]

Supplementary Information

Realisation of topological zero-energy mode in bilayer graphene in zero magnetic field

Janghee Lee[1], Kenji Watanabe[2], Takashi Taniguchi[2], and Hu-Jong Lee[1*]

[1]Department of Physics, Pohang University of Science and Technology, Pohang 790-784, Republic of Korea.

[2]National Institute for Materials Science, Namiki 1-1, Tsukuba, Ibaraki 305-0044, Japan.

*Correspondence and requests for materials should be addressed to H.-J. L. (email: hjlee@postech.ac.kr).

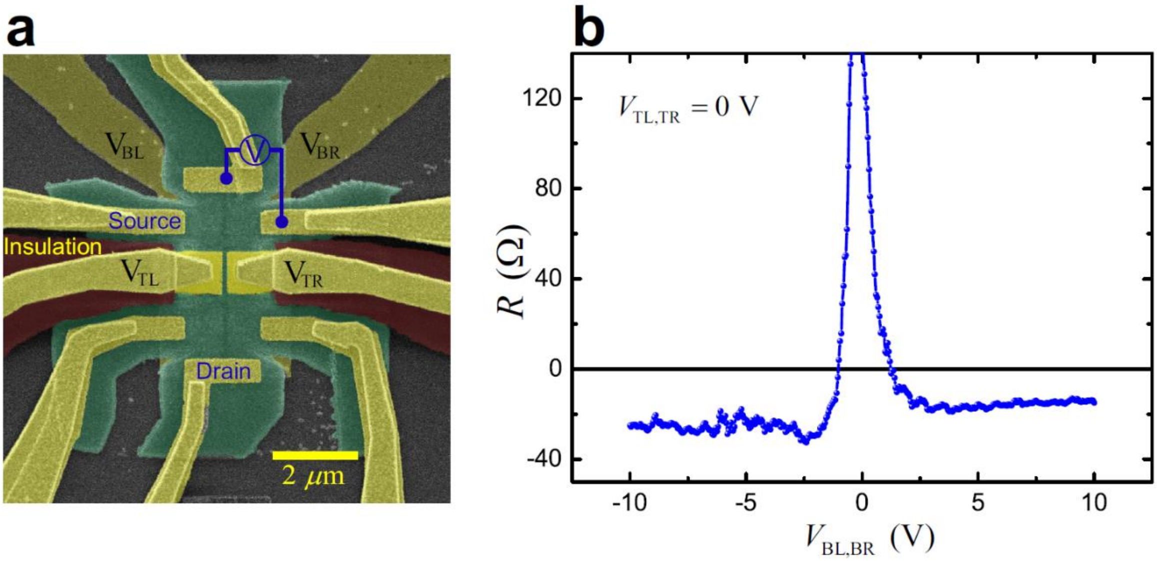

Supplementary Figure 1 | Negative resistance in a van der Pauw measurement configuration. (**a**) A van der Pauw measurement configuration. (**b**) $V_{BL,BR}$ dependence of resistance ($V_{TL,TR} = 0$) obtained from a van der Pauw measurement configuration, where the low-resistance range is focused. The negative resistance confirms the ballistic transport of carriers within the device.

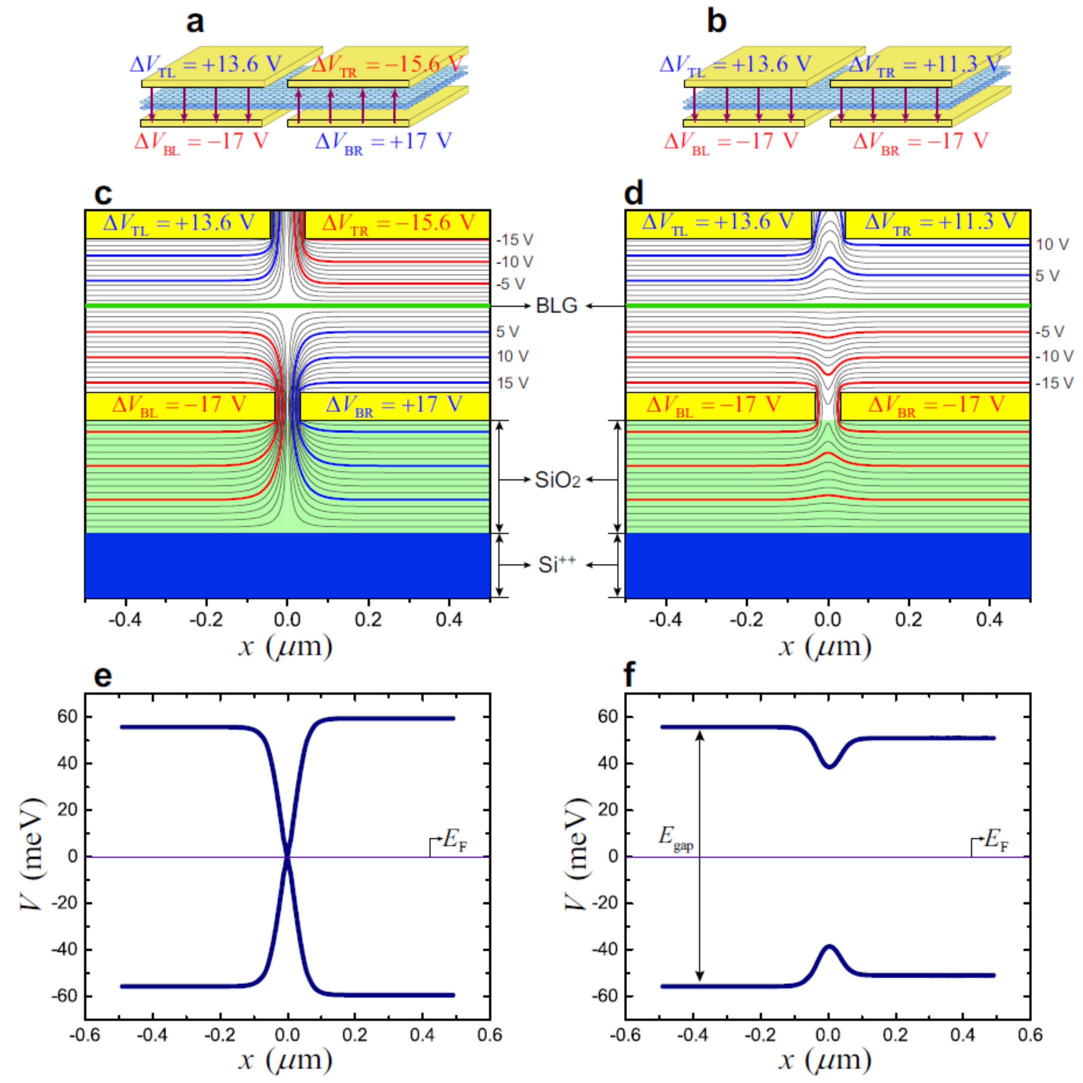

Supplementary Figure 2 | Numerical simulation for the kink-potential profiles. (**a**,**b**) Simulation models for the (**a**) asymmetric-gate [(**b**) symmetric-gate] configuration corresponding to the topmost (bottommost) curve in Fig. 4**a**. $\Delta V_{\mathrm{TL(TR)}} \equiv V_{\mathrm{TL(TR)}} - V_{\mathrm{T}}^{0}$ and $\Delta V_{\mathrm{BL(BR)}} \equiv V_{\mathrm{BL(BR)}} - V_{\mathrm{B}}^{0}$ where V_{T}^{0} and V_{B}^{0} are the CNP of the $V_{\mathrm{TL,TR}}$ and $V_{\mathrm{BL,BR}}$ determined from the conductance map in Fig. 2**a** (symmetric-gate configuration). (**c**,**d**) Kink-potential profiles calculated from models **a** and **b**. Lines are equipotential lines in steps of 1 V, and each 5 V step is denoted by thick blue (positive) and red (negative) lines. Thick green horizontal line near the centre indicates the BLG. (**e**,**f**) E_{gap} profiles calculated from the simulation results shown in **c** and **d**.

What is key to demonstrating the topological conduction in the BLG?

In this section, we discuss the key role played by the precise alignment of two (top and bottom) pairs of split gates in demonstrating the topological conductance along the kink-potential in the BLG. We further show that reducing the ratio of thickness between the bottom and top hBN is also important for demonstrating the transport characters related to the topological zero-energy mode in the BLG.

Supplementary Fig. 3**a** shows a false-coloured SEM image of another four-gate device with a schematic cross-section view of the dashed region in the SEM image. The centre of the spacing of the top split gate is misaligned from the centre of the bottom split gate by ~ 25 nm as shown in the schematic illustration in Supplementary Fig. 3**a**. As discussed in the main text, we define the symmetric-gate (asymmetric-gate) configuration as that where a pair of top and bottom split gates were connected to the respective voltage sources as shown schematically in Supplementary Fig. 3**b** (Fig. 3**c** and 3**d**). This device exhibited almost the same feature as that presented in the main text as plotted in Supplementary Fig. 3**b**, 3**c** and 3**d**. However, when $\vec{D}_{\mathrm{L}} \cdot \vec{D}_{\mathrm{R}} < 0$ [$\vec{D}_{\mathrm{L(R)}}$ is average displacement field in left (right) region] the resistance along the kink-potential boundary did not reach the value of the quantum resistance $h/(4e^2) \approx 6.45$ kΩ. Supplementary Fig. 3**e** (3**f**) shows slice plots corresponding to the dashed lines in Supplementary Fig. 3**c** (3**d**) for each value of V_{BR} and the inset is a magnified view of the shaded region. In the insets, the resistance of the device is at most 0.9 kΩ corresponding to about 28 e^2/h, which is much larger than that expected in the topological zero-energy mode. This implies that additional conducting channels were confined within the kink-potential at the corridor region as well as the topological zero-energy modes. From the numerical simulation, it is found that the small misalignment in the lateral position between two pairs of split gates induced topologically

trivial non-chiral conducting channels at the boundary between two insulating regions in the BLG.

Supplementary Fig. 4 presents the numerical simulation results for the gating effect in the dashed region in Supplementary Fig. 3**a**. All the lines in Supplementary Figs. 4**a** and 4**b** (upper panel in Supplementary Fig. 4**c**) are equipotential lines in steps of 1 V (0.02 V). For $\vec{D}_{\mathrm{L}} \cdot \vec{D}_{\mathrm{R}} > 0$, the misalignment barely affects the transport properties of the device as plotted in Supplementary Fig. 4**a**, where the equipotential lines are almost flat near the BLG. Therefore the insulating behaviour (large resistance) is clear at the upper-left and lower-right regions in Supplementary Fig. 3**b**. However, for $\vec{D}_{\mathrm{L}} \cdot \vec{D}_{\mathrm{R}} < 0$ (Supplementary Fig. 4**b**), the balance between top and bottom gates which makes D_n to vanish is broken near the kink-potential leading to non-zero value of D_n. Since D_n determines the total carrier density in the BLG induced by external gate voltages, the non-zero value of D_n near the kink-potential means that topologically trivial conducting channels exist in addition to the topological zero-energy modes.

As shown in the upper panel in Supplementary Fig. 4**c** (a magnified view of the dashed region in Supplementary Fig. 4**b**), the electric fields generated by positive voltages of V_{TR} and V_{BL} are stronger than those generated by negative V_{TR} and V_{BL}. Thus $D_n(x)$ becomes non-zero near the kink-potential area leading to accumulation of carriers near the corridor region. The lower panel in Supplementary Fig. 4**c** shows the variation of the local carrier density $n(x)$, calculated by the relationship $n = \frac{\varepsilon_0}{e} D_n$ with the same *x*-axis scale as that of the upper panel. A misalignment of ~25 nm between the top and bottom split gates can induce a large amount of carriers near the corridor region. This means that the excessive conductance of the device for $\vec{D}_{\mathrm{L}} \cdot \vec{D}_{\mathrm{R}} < 0$ shown in Supplementary Figs. 3**c** and 3**d** is a

result of the topologically trivial conducting channels induced by misalignment between the top and bottom split gates.

Supplementary Fig. 5**a** shows the numerical simulation results for estimating how $n(x)$ is affected by the degree of the misalignment (l_{mis}) between two pairs of split gates near the corridor. The topmost line is obtained from the simulation model with $l_{\mathrm{mis}} = 25\ \mathrm{nm}$, which corresponds to the device shown in Supplementary Fig. 3**a** (this line is the same as that in the lower panel in Supplementary Fig. 4**c**). As l_{mis} decreases (i.e., improving the alignment), $n(x)$ decreases gradually as shown in Supplementary Fig. 5**a**.

We also investigated the effect of thickness difference between the top and bottom hBN layers on $n(x)$ near the corridor region. Supplementary Fig. 5**b** shows $r_{d_{\mathrm{b}}/d_{\mathrm{t}}}$ (ratio of the thickness between the bottom and top hBN layers) dependence of the $n(x)$ for $l_{\mathrm{mis}} = 0$. The topmost line, which is plotted for comparison, is identical to the topmost line in Supplementary Fig. 5**a**, corresponding to the case of $l_{\mathrm{mis}} = 25\ \mathrm{nm}$ and $r_{d_{\mathrm{b}}/\mathrm{d_t}} = 1.9$. By lowering the value of $r_{d_{\mathrm{b}}/\mathrm{d_t}}$ close to one, residual $n(x)$ near the corridor region can be further reduced as shown in Supplementary Fig. 5**b**. Supplementary Fig. 5**c** shows the comparison for different $n(x)$ profiles corresponding to the devices in the main text and Supplementary Fig. 3**a**. In contrast to the device in Supplementary Fig. 3**a**, the simulation results for $n(x)$ of the device used in the main text shows significantly reduced values of *n* with small fluctuation near the corridor region. For the device used in the main text, l_{mis} is almost zero (within a few nm accuracy) and $r_{d_{\mathrm{b}}/\mathrm{d_t}} \approx 1.26$ ($r_{d_{\mathrm{b}}/\mathrm{d_t}} = 1.9$ for the device in Supplementary Fig. 3**a**). Therefore, a precise alignment of two pairs of split gates in combination with reducing the $r_{d_{\mathrm{b}}/\mathrm{d_t}}$ as close to 1 as possible is key to observing the topological conduction arising from the zero-energy mode.

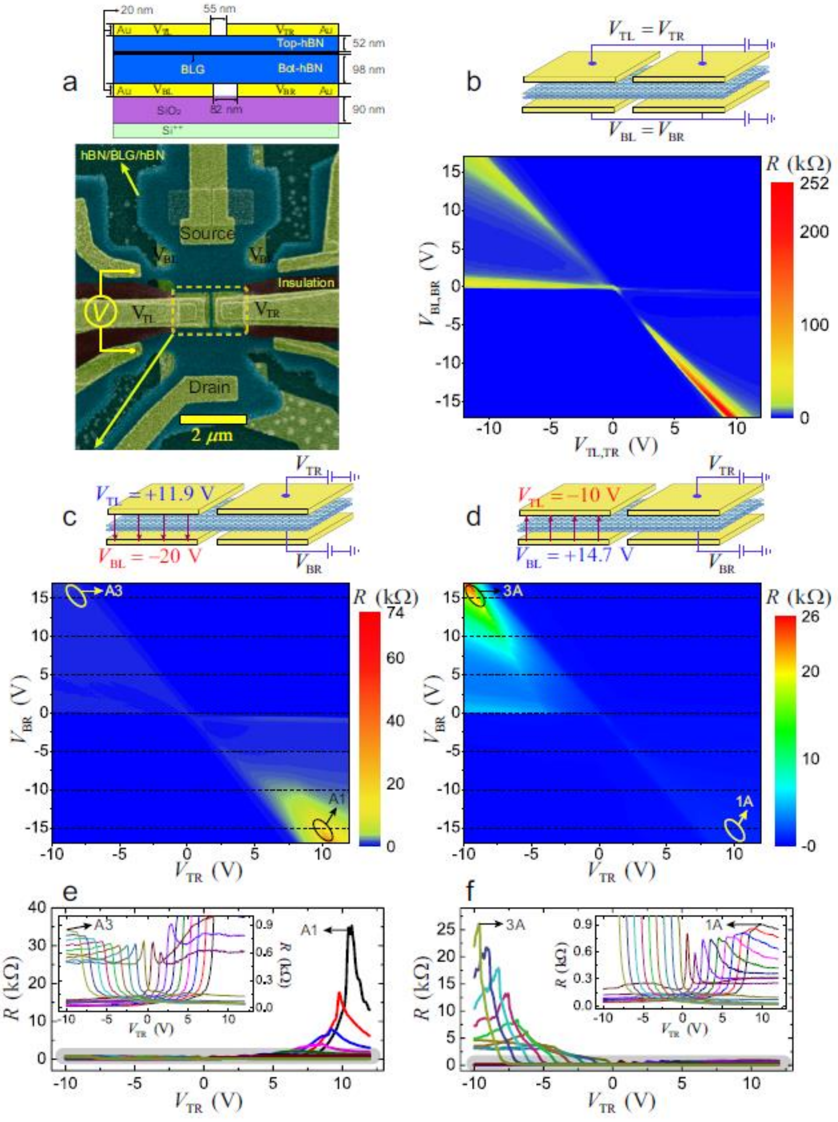

Supplementary Figure 3 | Symmetric- and asymmetric-gate configurations of a four-gate device. (**a**) A false-coloured SEM image of another four-gate device, whose two pairs (top and bottom) of split gates were misaligned by ~ 25 nm. Upper illustration shows the cross-sectional view of the dashed-boundary four-gated region in the SEM image. (**b**) $V_{TL,TR}$ and $V_{BL,BR}$ dependence of the resistance in colour code. (**c**,**d**) V_{TR} and V_{BR} dependence of the resistance in colour code. Upper illustrations in **b**, **c** and **d** show schematic measurement configurations corresponding to resistance maps in **b**, **c** and **d**, respectively. (**e**,**f**) Slice traces corresponding to dashed lines in the conductance maps in **c** and **d**. Insets are magnified views of the shaded regions (low resistance region).

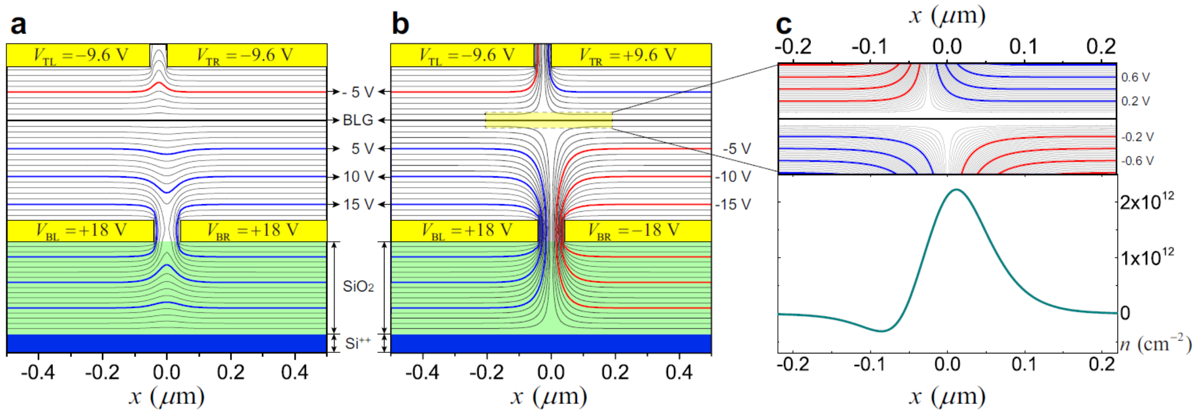

Supplementary Figure 4 | Simulation results for the misalignment effect. (**a**,**b**) Simulation results for the potential profiles in a 25-nm-misaligned four-gate device for (**a**) $\vec{D}_{\mathrm{L}} \cdot \vec{D}_{\mathrm{R}} > 0$ and (**b**) $\vec{D}_{\mathrm{L}} \cdot \vec{D}_{\mathrm{R}} < 0$. Lines are equipotential lines in steps of 1 V. Each 5 V step is denoted by thick blue (positive) and red (negative) lines. (**c**) Magnified view (upper panel) of the dashed region in **b** and the induced local carrier density profile (lower panel) in the same *x*-axis scale. All the lines in upper panel are equipotential lines in steps of 0.02 V. Each 0.2 V step is denoted by thick blue (positive) and red (negative) lines. The local carrier density profile in lower panel was calculated from the relationship $n = \frac{\varepsilon_0}{e} D_n$. Positive (negative) value means the electron-type (hole-type) carriers.

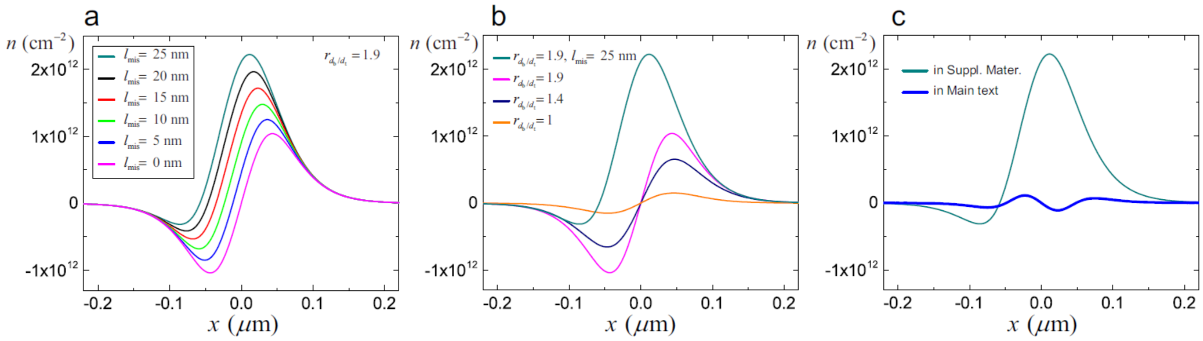

Supplementary Figure 5 | Simulation results for the local carrier density profiles. (**a**) Simulation results for the local carrier density profile $n(x)$ near the corridor region, obtained using the model corresponding to the device in Supplementary Fig. 3**a** for each l_{mis} (degree of the misalignment between top and bottom split gates). (**b**) The $r_{d_{\text{b}}/d_{\text{t}}}$ (thickness ratio between the bottom and top hBN layers) dependence of $n(x)$ for $l_{\text{mis}} = 0$. The topmost curve is identical to the topmost one in **a**. (**c**) Comparison of $n(x)$ between two devices (one in the main text and another in Supplementary Fig. 3**a**).

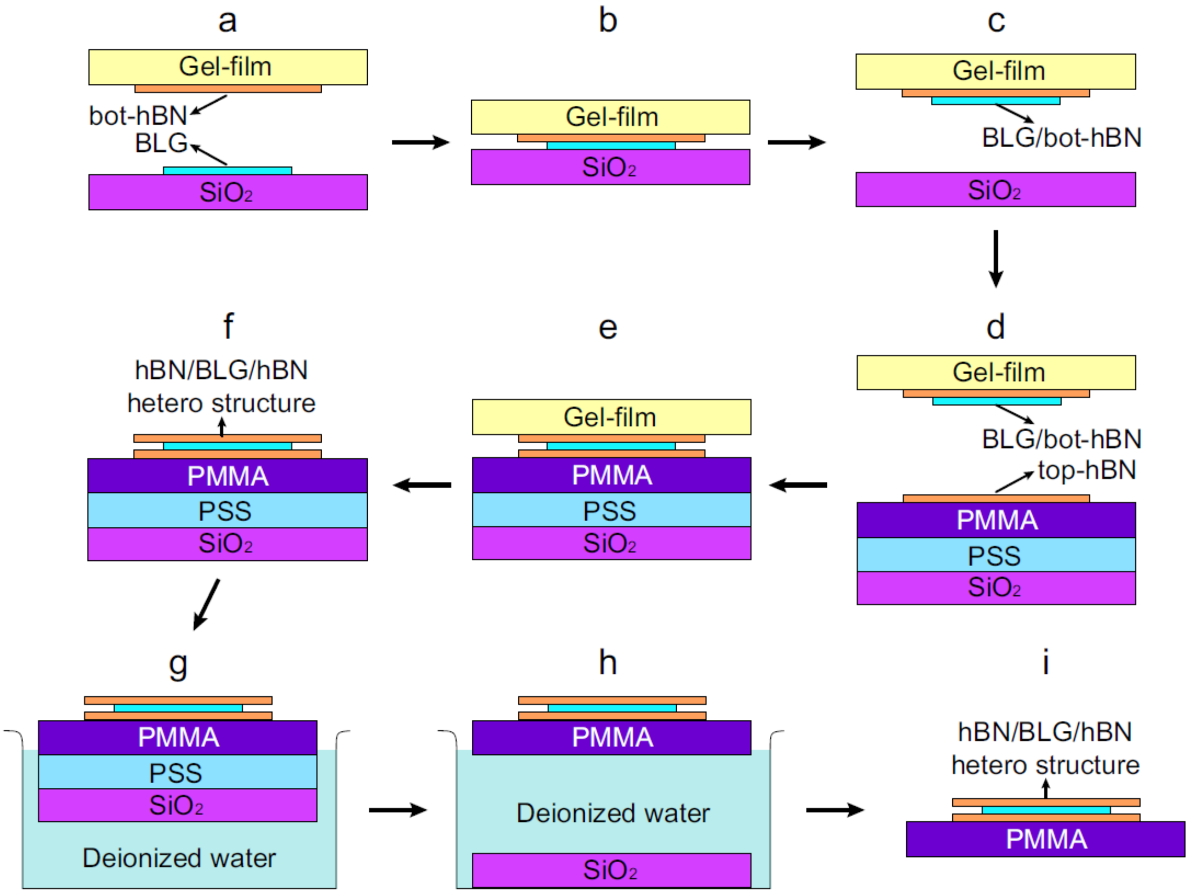

Supplementary Figure 6 | Stacking and transferring procedure. Schematic illustrations of the (**a-f**) stacking and (**g-i**) transferring procedure. Details are described in the Method section.

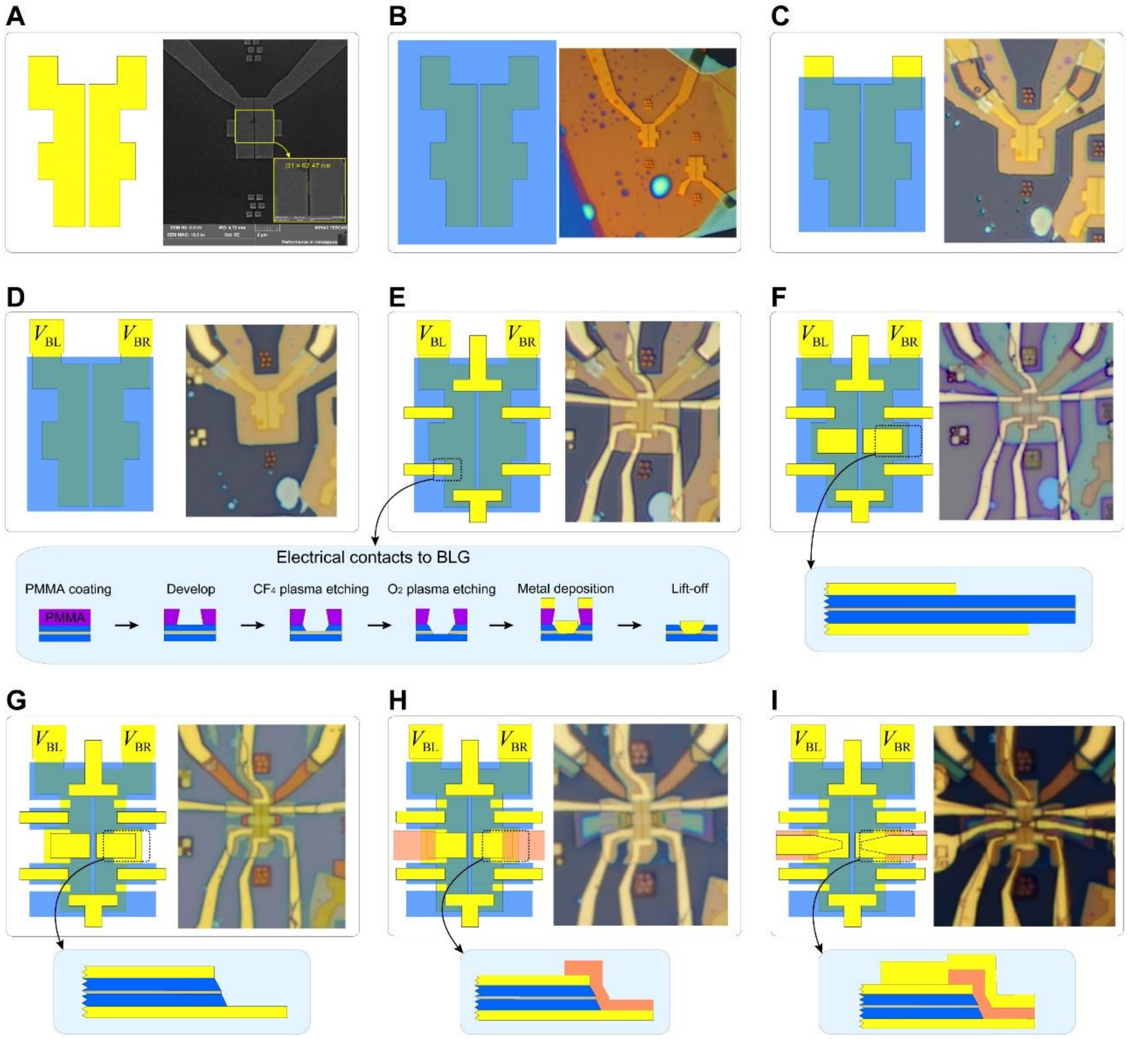

Supplementary Figure 7 | Device design and patterning procedure. Schematic illustrations with the SEM or optical microscopy (OM) images corresponding to each step. (**a**) The split bottom gates. (**b**) OM image of the hBN/BLG/hBN heterostructure after transferring it onto the split bottom gates. (**c**) O_2/CF_4 plasma etching to outline the device while exposing the bottom-gate contacts. (**d**) Connecting the bottom-gate extension leads (V_{BL} and V_{BR}). (**e**) Electrical contacts to the BLG. Highlighted view shows the procedure of making the electrical contacts to the BLG. (**f**) Two split top-gate pads. (**g**) O_2/CF_4 plasma etching to define the device configuration. (**h**) Double insulating layers of Al_2O_3 (120 nm) and cross-linked PMMA (~130 nm). (**i**) Top-gate extension leads. The cross-sectional view highlights the dotted-box region in the panel from **f** to **i**. See Method section for details.

Transport properties of the dual-gated bilayer graphene.

In this study, another type of device (two-gate device) was fabricated without splitting the top and bottom gates by using the same method as that described in Method section in the main text. Therefore, the two-gate device is the same as conventional dual-gated devices with a top (V_{top}) and bottom (V_{bot}) gates.

Supplementary Fig. 8**a** is a false-coloured scanning electron microscopy (SEM) image of the two-gate device. As described in the main text, we define the displacement field as follows:

$$\vec{D}_{top} = -\varepsilon_{hBN}\frac{V_{top}-V_{top}^{0}}{d_{top}}\hat{z} \quad , \vec{D}_{bot} = \varepsilon_{hBN}\frac{V_{bot}-V_{bot}^{0}}{d_{bot}}\hat{z} \qquad (1)$$

where, ε_{hBN} is the dielectric constant of hBN[21] (~3.9), d_{top} (d_{bot}) is the thickness of the top (bottom) hBN, V_{top}^{0} (V_{bot}^{0}) is the charge neutrality point (CNP) of V_{top} (V_{bot}), and $\hat{z}$ is the unit vector normal to the BLG sheet. Then the magnitude of the average displacement field, $\left|\vec{D}_{avg}\right| = \left|\frac{\vec{D}_{top}+\vec{D}_{bot}}{2}\right|$, determines the size of the band gap (E_{gap}) and the total carrier density in the BLG can be estimated by $n = \frac{\varepsilon_0}{e}\left(\vec{D}_{bot} - \vec{D}_{top}\right)\cdot\hat{z} \equiv \frac{\varepsilon_0}{e}D_n$.

The V_{top} and V_{bot} dependence of the resistance of the two-gate device exhibits a typical feature of the dual-gate BLG as presented in Supplementary Fig. 8**b** for each curve and Fig. 8**c** in a color-coded plot as a function of $\vec{D}_{top}\cdot\hat{z}\ (\equiv D_{top})$ and $\vec{D}_{bot}\cdot\hat{z}\ (\equiv D_{bot})$. At the point denoted by 'α' in Supplementary Fig. 8**c**, $D_{top} = D_{bot} = 0$. Thus, $E_{gap} = 0$ and E_F is located at the CNP $(D_n = 0)$ as shown in Supplementary Fig. 8**d** with the same notation of 'α'. Note that '$D_n = 0$' does not mean '$n = 0$' at α. Because there are residual carriers even though the E_F is located at the CNP. Along the dashed line in Supplementary Fig. 8**c**, the $\left|\vec{D}_{avg}\right|$ is kept at zero, thus $E_{gap} = 0$ as illustrated in Supplementary Fig. 8**d** with the same notations of δ, α and β. On the other hand, D_n increases from negative to

positive with a sequence of ‘δ < 0’, ‘α = 0’ and ‘β > 0’ as illustrated in Supplementary Fig. 8**d**. The variation of the E_{gap} takes place along the parallel lines to the solid line in Supplementary Fig. 8**c**, where D_n is kept at constant value. For example, the E_{F} remains in the middle of the E_{gap} along the solid line in Supplementary Fig. 8**c**, however, the $|\vec{D}_{\text{avg}}|$ increases as one moves away more from the point α. Therefore, the large resistances at σ and γ are caused by a formation of the band gap with E_{F} in the middle of the E_{gap}.

Supplementary Fig. 9**a** shows current-voltage (*I-V*) characteristics obtained from the two-gate device at 4.2 K along the solid line in Supplementary Fig. 8**c**. At point α, BLG becomes a gapless semiconductor $(E_{\text{gap}} = 0)$, thus the *I-V* curve is almost linear as shown in Supplementary Fig. 9**a**. However, the transport gap $(E_{\text{gap}}^{\text{tr}})$ in *I-V* curves increases as the $|D_{\text{avg}}|$ increases (from α to σ or γ). From the linear extrapolation in high current region of the *I-V* curves, we roughly estimated the $E_{\text{gap}}^{\text{tr}}$ as shown in Supplementary Fig. 9**b** and the results are summarized in Supplementary Fig. 9**d**. We also measured temperature (*T*) dependence of the resistance (Supplementary Fig. 9**c**) for each point along the solid line in Supplementary Fig. 8**c**. When the E_{F} lies in the middle of E_{gap}, the resistance increases exponentially with decreasing *T* as $R(T) \propto \exp(E_{\text{gap}}/2k_BT)$, where k_{B} is Boltzmann’s constant. Then the E_{gap} can be extracted from the linear plot of $\ln(R)$ vs $1/T$ (Arrhenius law). The inset in Supplementary Fig. 9**c** shows the Arrhenius-law fitting and the results are plotted in Supplementary Fig. 9**d**. The solid line in Supplementary Fig. 9**d** is $|D_{\text{avg}}|$ dependence of the E_{gap} calculated by self-consistent tight-binding model in previous study[12]. The red circles are $E_{\text{gap}}^{\text{tr}}$ (transport gap) estimated from *I-V* curves and the blue circles are $E_{\text{gap}}^{\text{th}}$ (thermal activation gap) extracted from the Arrhenius fit to the *T*

dependence of resistance. The discrepancy between $E_{\text{gap}}^{\text{th}}$ and $E_{\text{gap}}^{\text{tr}}$ can be explained as followings.

The method described in Supplementary Fig. 9**b** has an uncertainty (overestimating) in determining E_{gap} from $E_{\text{gap}}^{\text{tr}}$, unless the T is zero and there are no in-gap impurity bands. There is also an ambiguity in extracting $E_{\text{gap}}^{\text{th}}$ from the $\ln(R)$ vs $1/T$. $E_{\text{gap}}^{\text{th}}$ is determined from the slope of $\ln(R)$ vs $1/T$ at high T region (or at low region of $1/T$). And only the resistance of the dual-gated region (R_{dual}) in the BLG should be included in $\ln(R)$. However, because the total resistance was measured by quasi four-probe measurement as shown in Supplementary Fig. 8**a**, the resistance of the region in the BLG that was not top-gated (R_{offset}) was included in the total resistance. At high T region, R_{dual} is not very large compared to R_{offset}, thus the $E_{\text{gap}}^{\text{th}}$, which is extracted from the slope of $\ln(R)$ vs $1/T$, can be underestimated compared to E_{gap}. It is worth to note that the previous results on the $E_{\text{gap}}^{\text{tr}}$ were at most 1 ~ 2 mV even at 50 mK. However, even though there is an uncertainty, our result on $E_{\text{gap}}^{\text{tr}}$ is at least one order of magnitude larger than those in previous studies. This means that the residual conductance problem in gapped BLG may be caused by impurity-induced in-gap states rather than by the topological edge channels. In other words, preparing a high quality BLG (e.g., encapsulation of the BLG as in this study) may lead to large $E_{\text{gap}}^{\text{tr}}$ comparable to the optical measurement results. Referring to the results of our two-gate device, dual-gated BLG samples of earlier studies, prepared on a SiO_2 substrate and exposed to various chemical materials, which subjected to the formation of charged impurities or defects.

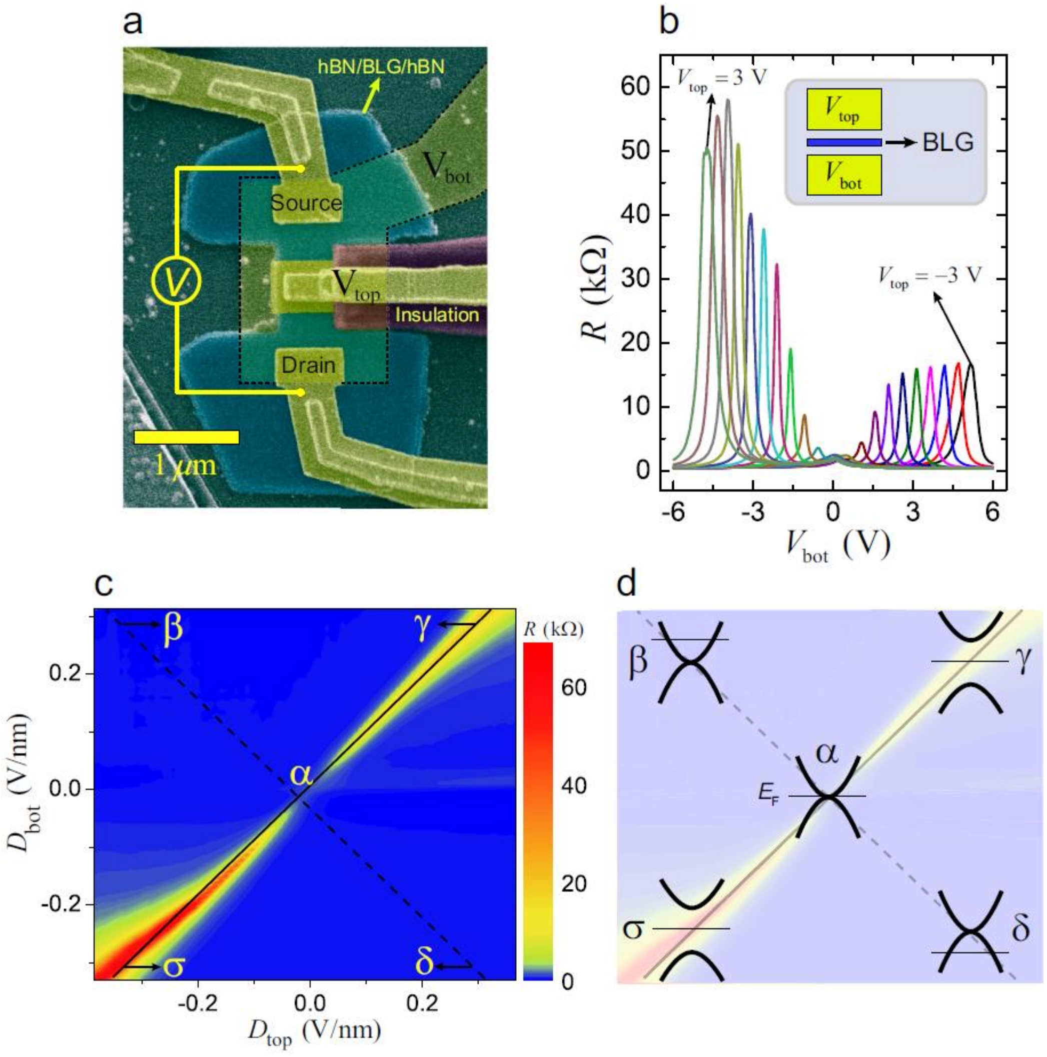

Supplementary Figure 8 | Transport properties of a dual-gated BLG. (**a**) A false-coloured SEM image of a two-gate device. The dashed line is the boundary of the bottom gate electrode, which is located under the hBN/BLG/hBN heterostructure. (**b**) The V_{bot} dependence of the resistance for each value of V_{top} from -3 V to 3 V. Inset is a schematic illustration of the cross-section of the two-gate device. (**c**) Colour-coded plot of the D_{top} and D_{bot} dependence of the resistance, which is generated from **b**. (**d**) Schematic illustration of the low-energy bands in BLG corresponding to each point [α, β, γ, δ and σ in **c**]. Horizontal lines indicate the Fermi energy E_F.

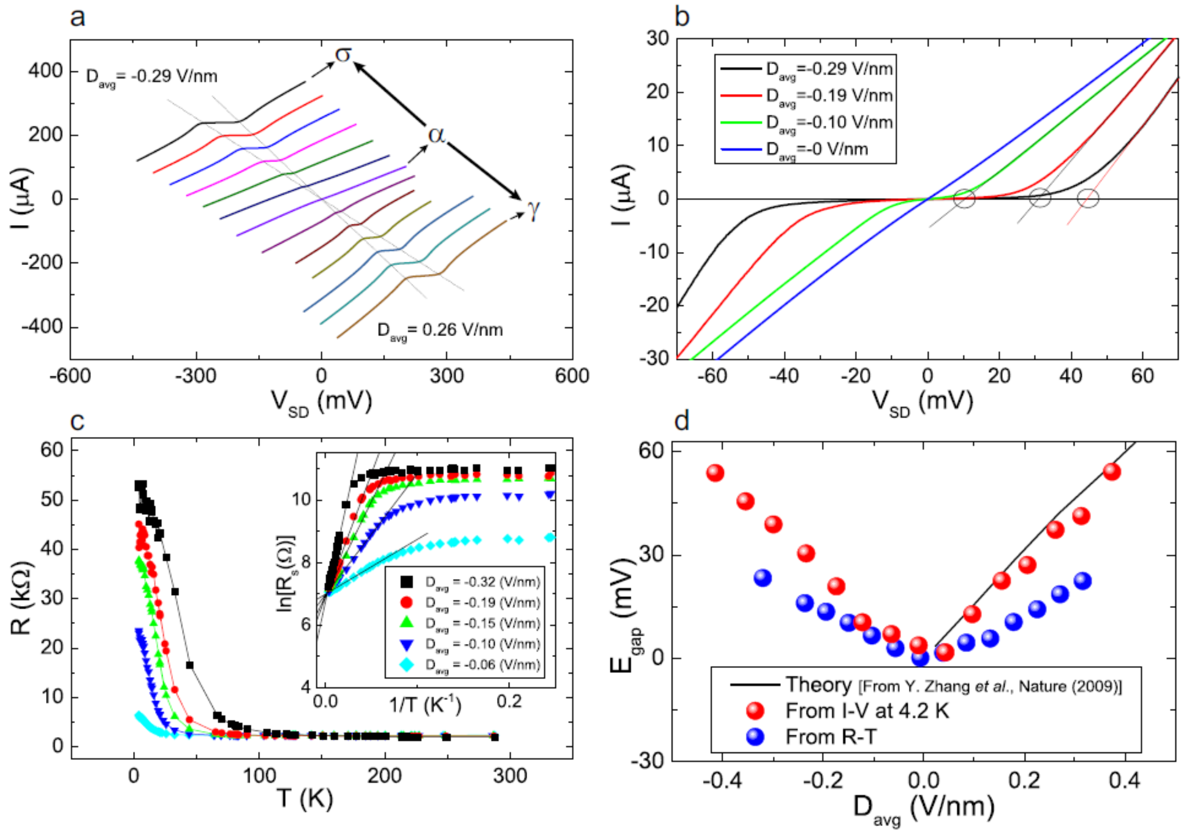

Supplementary Figure 9 | Transport gap and thermal excitation gap of a two-gate device. (**a**) *I*-*V* curves for each D_{avg} from –0.29 V/nm (upper left) to 0.28 V/nm (lower right) along the solid line in Supplementary Fig. 8**c**. Crossing dotted lines are guides to eyes for the transport gap $\left(E_{\mathrm{gap}}^{\mathrm{tr}}\right)$. All the curves are shifted for clarity. (**b**) *I*-*V* curves for selected D_{avg}. The $E_{\mathrm{gap}}^{\mathrm{tr}}$ (circles) was determined by choosing the intercept of the extrapolation in the high current region. (**c**) Temperature dependence of resistance for each selected D_{avg} along the solid line in Supplementary Fig. 8**c**. Inset: Arrhenius plot to determine the thermal excitation gap $\left(E_{\mathrm{gap}}^{\mathrm{th}}\right)$. (**d**) Comparison of $E_{\mathrm{gap}}^{\mathrm{th}}$ and $E_{\mathrm{gap}}^{\mathrm{tr}}$ with the theoretical calculation[12].
